# Exclusive enteral nutrition containing transforming growth factor‐β improves intestinal barrier function in a colitis mouse model

**DOI:** 10.1002/jpn3.70517

**Published:** 2026-07-21

**Authors:** Kawthar Boumessid, Vickie Lacroix, Ekaterina Ovtchinnikova, Sophie Thenet, Marie Carriere, Frédérick Barreau, Emmanuel Mas

**Affiliations:** ^1^ INSERM UMR1220, INRAE, ENVT University of Toulouse Toulouse France; ^2^ Gastroenterology, Hepatology, Nutrition, Diabetes and Inborn Errors of Metabolism Department, Children Hospital Toulouse University Hospital Toulouse France; ^3^ UMR 938, Research Center of Saint‐Antoine Sorbonne University, INSERM Paris France; ^4^ EPHE PSL University Paris France; ^5^ SyMMES University of Grenoble, CEA, INP, CNRS Grenoble France

**Keywords:** Crohn's disease, intestinal organoids, intestinal permeability

## Abstract

**Objectives:**

Exclusive enteral nutrition (EEN) is the first‐line treatment for pediatric Crohn's disease, but its mechanisms of action remain poorly understood. Our aim was to identify the mechanisms that could explain the anti‐inflammatory effects of EEN, studying the nutritional composition and transforming growth factor‐β (TGF‐β) effects, in a mouse model of colitis.

**Methods:**

Mice were treated with dextran sulfate sodium (DSS) to induce colitis. After DSS treatment, we compared two enteral nutrition formulas, and we evaluated the effect of TGF‐β itself on clinical and microscopic inflammation, and intestinal permeability, by TGF‐β‐supplementation, ‐inhibition, or ‐deletion. Colonic crypts from DSS and EEN mice were cultured and their cellular properties were analyzed.

**Results:**

Both EEN formulas improved weight recovery and disease activity index. In contrast, EEN mice treated with TGF‐β formula presented faster weight recovery and decreased inflammatory parameters, with a normalized intestinal permeability, suggesting gut restitution and functionality. These functional improvements were not found in the absence of TGF‐β in the formulas. Finally, organoids from colonic crypts treated with Modulen IBD® containing TGF‐β showed enhanced survival and re‐epithelialization capacity.

**Conclusions:**

Both EEN formulas have anti‐inflammatory properties based on their nutritional composition. However, TGF‐β plays a significant role in intestinal functional restitution.

## Abstract (highlights)

**What is Known:**

- Exclusive enteral nutrition is recommended in Crohn's disease.
- Its mechanism of action is still unknown.

**What is New:**

- The nutritional matrix and transforming growth factor‐β (TGF‐β) have anti‐inflammatory properties.
- TGF‐β restores a functional intestinal epithelium.

## Graphical abstract

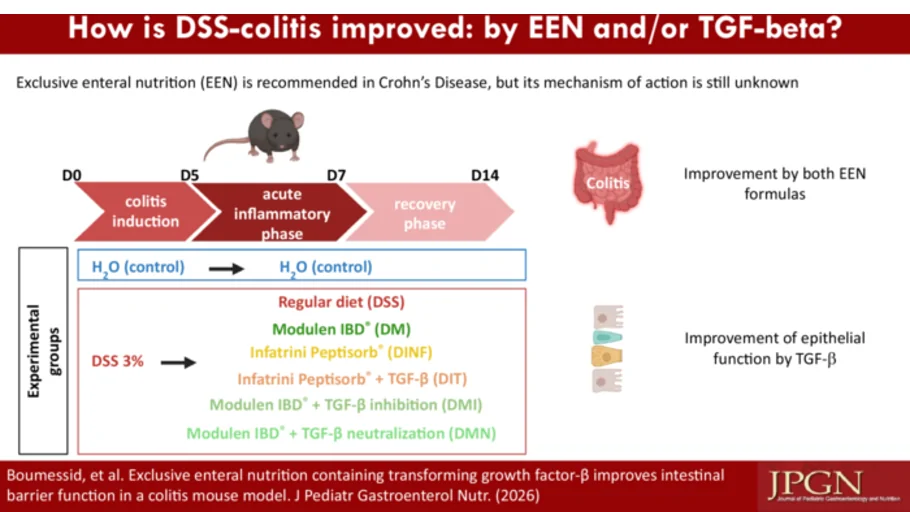

## 1 INTRODUCTION

Crohn's disease (CD), a type of inflammatory bowel disease (IBD), represents a worldwide health issue since its incidence is continuously increasing. CD is characterized by chronic relapsing inflammation of the digestive tract, with discontinuous and deep lesions. It is due to both genetic and environmental factors. Although not as important as environmental factors, CD also has a strong genetic component. The pathophysiology of CD results from an abnormal immune response associated with an altered intestinal microbiota.^1^, ^2^ Although the causal link(s) between these alterations is unknown, a barrier defect evidenced by an increased intestinal permeability is involved in the genesis and maintenance of this abnormal communication, leading to inflammation and epithelium alterations.^3^ The integrity of the intestinal epithelium is crucial and is ensured by intestinal stem cells (ISCs), which allow the epithelium renewal in 3–5 days.^4^ ISC are endowed with self‐renewal and multipotency capacities, by generating progenitors that differentiate into mature and functional cells. In IBD, a default of the epithelial renewal has been documented.

Main CD treatments target the immune system or the inflammatory process such as anti‐inflammatory drugs (steroids, 5‐aminosalicylic acid), immune modulators, and biologics.^5^ Although these treatments improve symptoms, they are not sufficient to avoid a relapse. Exclusive enteral nutrition (EEN) is effective, with up to 86% clinical remission, associated with mucosal healing.^5^, ^6^ Thus, EEN is the first‐line treatment for pediatric CD in case of non‐stricturing or penetrating disease.^7^ Besides its clinical efficiency, EEN permits mucosal healing.^5^ By far, mucosal healing represents the best criteria for long‐term clinical remission, which makes it a therapeutic end‐point for CD. While many mechanisms could explain these results,^5^ feeding with a unique formula could reduce intestinal inflammation through different mechanisms, but the effect could be also driven by the transforming growth factor‐β (TGF‐β). In IBD, decreasing intestinal inflammation, and permeability, as well as restoring the gut barrier mediated by TGF‐β is hypothetical. Meanwhile, TGF‐β is one of the mediators that regulate the epithelium renewal.^8^ TGF‐β is involved in the first phase of mucosal healing, also called “restitution,” which is based on the redistribution of existing cells to fill in the ulcerated areas. Moreover, TGF‐β is also described to maintain the integrity of tight junctions, prevent Goblet cells' depletion, and is involved in immune regulation.^8^, ^9^, ^10^, ^11^ TGF‐β production and/or signaling are dysregulated in IBD.^8^, ^11^, ^12^ Thus, we aimed to investigate the mechanisms that could explain the anti‐inflammatory effects of EEN, studying the nutritional composition and TGF‐β effects, in a mouse model of colitis.^12^

## 2 METHODS

### 2.1 Ethics statement

Animal experimentations were approved by the ethical committee (Comité d'Ethique et d'Expérimentation animale 122), and performed under the protocols “APAFIS#28278‐2020111810056802, and 2020120418538745.”

### 2.2 Mice

C57BL/6Nrj wild‐type and *Nod2* ^*KO*^ male mice of 12 weeks of age were hosted in the animal facility of Toulouse Purpan.^13^

### 2.3 Experimental procedures

The study was conducted from October 2019 to December 2022. Mice were treated with dextran sulfate sodium (DSS) at 3% during 5 days to induce a colitis, and then DSS was replaced with regular water (DSS group). Disease progression was monitored daily until D14. Instead of regular food, other mice received EEN from D5, until the sacrifice day, either at D7 or D14. We used two formulas *ad libitum*, a naturally TGF‐β‐rich formula (Modulen‐IBD® [*Nestlé*], 8.0 ± 0.6 ng/mL) (DSS Modulen‐IBD® [DM] group) and another formula that does not contain TGF‐β (Infatrini‐Peptisorb® [*Nutricia*]) (DSS Infatrini‐Peptisorb® [DINF] group, 0 ng/mL). We performed TGF‐β neutralization (DSS Modulen‐IBD® + TGF‐β neutralization [DMN] group, 0 ng/mL), TGF‐β signaling inhibition (DSS Modulen‐IBD® + TGF‐β signaling inhibition [DMI] group), and TGF‐β2 addition (DSS Infatrini‐Peptisorb® + TGF‐β addition [DIT]; group, 7.8 ± 0.5 ng/mL) (Figure S1). More details concerning the pharmacological treatments are available in supplementary material.

### 2.4 Clinical observations

To assess colitis severity, body weight was monitored daily in order to establish a disease activity index (DAI).^14^ More information is available in supplementary material.

### 2.5 Macroscopic analysis

A macroscopic inflammation score was determined by analyzing colon appearance (colonic length, thickness, and edema). More details are available in supplementary material.

### 2.6 Histological analysis

The entire colons were collected, enrolled, fixed in formol overnight, then in 70% ethanol at 4°C. More details are available in supplementary material.

### 2.7 Paracellular permeability

Distal colonic samples were mounted in Ussing chambers.^15^ The mucosal to serosal flux was measured with 4 kDa fluorescein isothiocyanate‐Dextran to assess the paracellular permeability.^16^

### 2.8 Colonic organoid culture

After sacrifice, colons were opened longitudinally and washed in PBS. Organoid culture is described in ementary material.

### 2.9 RNA extraction, reverse transcription, and quantitative PCR

RNA from colonic crypts were extracted by Trizol reagent.^17^ RNA extraction, reverse transcription, and PCR methodologies are described in supplementary material and Table S1.

### 2.10 DNA methylation

The levels of 5‐methyl deoxycytosine were measured by high‐performance liquid chromatography‐mass spectrometry. DNA methylation is described in supplementary material.

### 2.11 Statistical analysis

Statistical analysis was done with GraphPad Prism v9. For all the analyses, the Gaussian distribution was tested by the Kolmogorov–Smirnov test. For two‐group comparisons, an unpaired parametric *t*‐test assuming the Gaussian distribution was applied. One‐way analysis of variance (ANOVA) statistics with Bonferroni correction were performed for multiple comparisons. A two‐sided *p*‐value < 0.05 was considered statistically significant. All data are presented as mean ± SEM. *p*‐Values are: *p < 0.05; **p < 0.01; ***p < 0.001; ****p < 0.0001.

## 3 RESULTS

### 3.1 EEN with a CD‐specific formula containing TGF‐β has anti‐inflammatory properties and promotes gut healing

The first step consisted of developing an experimental model reproducing the anti‐inflammatory properties of EEN, as reported in clinical studies and by our clinical data (Figure S2). Indeed, CD children treated by EEN with Modulen‐IBD® have shown significant health improvements, evidenced by body weight increase, C‐reactive protein (CRP) level decrease, and albumin level increase. Thus, clinical and inflammatory parameters were monitored in DSS mice treated or not with TGF‐β formula (DM and DSS, respectively). DSS mice lose weight from D7 to D10, and exhibit an elevated DAI, evidencing colitis development (Figure 1A,B). Moreover, macroscopic inflammation, determined by colonic shortening (Figure 1C) and thickening (Figure 1D), was accompanied by a high macroscopic inflammatory score at D7, and D14 (Figure 1C–E). DM decreased colitis‐induced weight loss, DAI, macroscopic inflammation score, and edema score (Figure 1A–F). However, colonic length did not change between DM and DSS groups (Figure 1C). DM decreased colonic thickness at D14, whereas there was no difference at D7 (Figure 1D). At the microscopic level, DSS induced a crypt shortening, and a decrease in the cell number per crypt (Figure 1G,H). It also increased pro‐inflammatory cytokine CxCl1/KC expression, which was improved by DM at D7 (Figure 1I). The histological score was attenuated by DM, specifically crypt irregularities, hyperplasia, and erosions (Figure S3). DM raised the total number of cells per crypt, and the crypt length at D7 but not at D14 (Figure 1G,H). Furthermore, DM normalized the high colonic permeability induced by DSS (Figure 1J).

**Figure 1:**
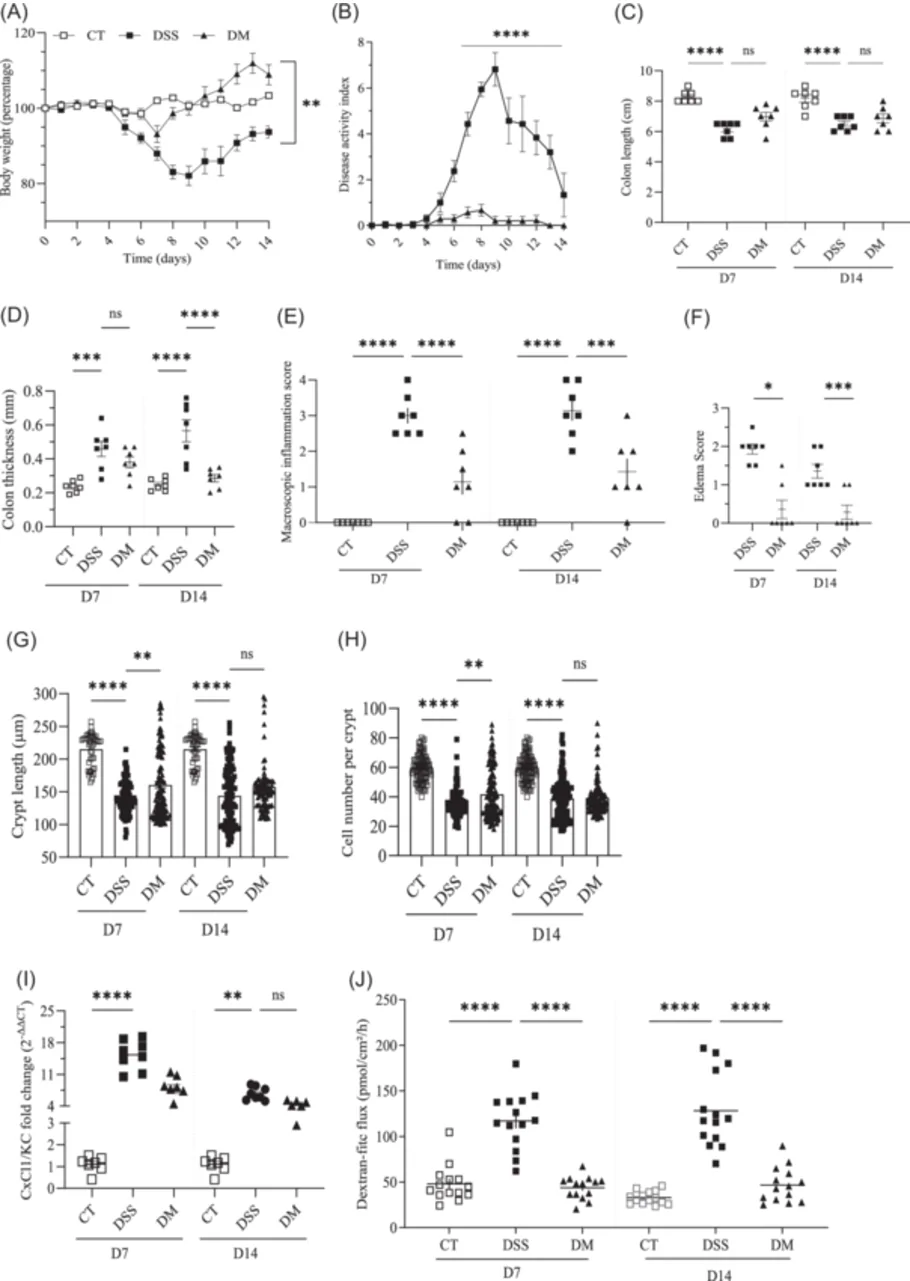
Exclusive enteral nutrition with a TGF‐β‐rich formula has anti‐inflammatory properties, and promotes intestinal healing in a mouse model of colitis. (A) Body weight monitoring of CT, DSS, DM (DSS + Modulen‐IBD®) treated mice (legend at the top). (B) DAI during DSS procedure (same legend as A). (C) Colon length at day 7 (D7) and D14 expressed in cm. (D) Colon thickness at D7 and D14 expressed in mm. (E) Macroscopic inflammatory score at D7 and D14. (F) Edema scoring of DSS and DM mice at D7 and D14. (G) Crypt length measured in µm at D7 (minimum of 44 crypts per sample, two samples per condition). (H) Total cell number per crypt at D7 (minimum of 44 crypts per sample, two samples per condition). (I) mRNA relative expression of *CxCl1/KC* from crypts of CT, DSS, and DM mice (*n* > 4). (J) Dextran‐FITC flux representing intestinal permeability performed in duplicate, each point equivalent to a duplicate. Results are expressed as mean ± SEM; *n* = 7 mice per group. *p*‐values: *p < 0.05; **p < 0.01; ***p < 0.001; ****p < 0.0001 versus DSS group. CT, control; DAI, disease activity index; DSS, dextran sulfate sodium; FITC, fluorescein isothiocyanate; TGF‐β, transforming growth factor‐β; SEM, standard error of the mean.

We wanted to validate these results in a mouse model predisposed to develop CD. In Humans, patients harboring *NOD2* mutations have an increased risk of developing CD.^18^ Interestingly, *Nod2* ^−/−^ mice treated by EEN with Modulen‐IBD® exhibited a similar improvement of the colitis than those observed in WT mice including body weight, DAI, macroscopic inflammation, colon length and thickness, and permeability (Figure S4).

### 3.2 Both EEN formulas improve colitis while the presence of TGF‐β is required to normalize intestinal permeability

The nutritional matrix, as well as TGF‐β contained in a few formulas (Modulen‐IBD® and Santactiv Digest®) could play an important role that we studied (Table S2). The Modulen‐IBD® formula contained 8 ng/mL of TGF‐β2 whereas it was undetectable in Infratrini Peptisorb® (Figure S1). EEN itself, with (DM) or without TGF‐β (DINF) decreased clinical parameters of inflammation (Figure 2). The supplementation with TGF‐β2 (DIT) and its neutralization (DMN) did not affect weight loss, DAI, or colon length (Figure 2A–C). In contrast, only the naturally TGF‐β‐rich formula (DM) decreased the colon thickness at D14 (Figure 2D), macroscopic scoring (Figure 2E), edema score (Figure 2F), and restored a normal permeability (Figure 2G). Some of these beneficial impacts were lost when TGF‐β was neutralized (DMN), including macroscopic and edema score, and permeability (Figure 2E–F). On the other hand, supplementation of the other formula with TGF‐β (DIT) tended to decrease macroscopic inflammatory score and edema, and even completely abolished in some mice at D14, as observed for DM (Figure 2E–F). Regarding colonic permeability, formula without TGF‐β (DMN/DINF) did not mitigate hyperpermeability induced by DSS, whereas its supplementation (DIT) normalized it (Figure 2G). These results suggest that both EEN formulas and TGF‐β play a role in the anti‐inflammatory properties of EEN. However, TGF‐β seems to play a major role in the epithelium functionality by restoring gut permeability. Similarly, inhibition of TGF‐β signaling pathway during EEN (DMI) confirmed these results (Figure 3).

**Figure 2:**
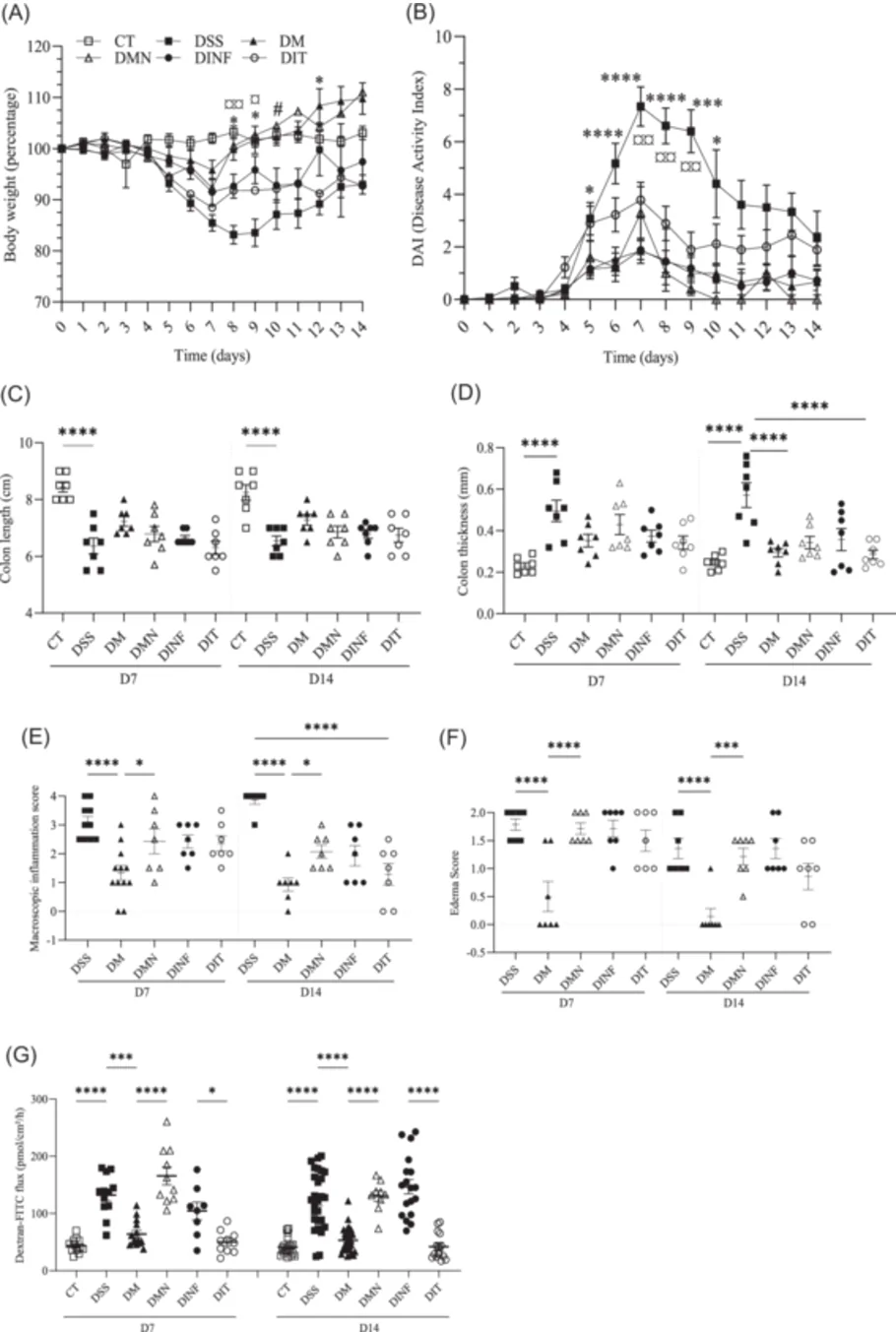
Exclusive enteral nutrition formulas and TGF‐β are necessary to restore intestinal homeostasis in a mouse model of colitis. (A) Body weight evolution of CT (control), DSS, DM (DSS + Modulen‐IBD®), DMN (DSS + Modulen‐IBD® TGF‐β neutralization), DINF (DSS + Infatrini‐Peptisorb®), DIT (DSS + Infatrini‐Peptisorb® + TGF‐β) (legend at the top) (*n* > 10). Statistical analysis: * (DINF vs. DSS), # (DINF vs. DM), ¤ (DIT vs. DM). (B) DAI during experimentation (same legend as A). (C) Colon length in cm at D7 and D14. (D) Colon thickness in mm at D7 and D14. (E) Macroscopic inflammation score at D7 and D14. (F) Edema scoring at D7 and D14. (G) Intestinal permeability performed in duplicate, each point equivalent to a duplicate. Data are expressed as mean ± SEM; *n* = 7 mice per group. *p*‐Values: *p < 0.05; **p < 0.01; ***p < 0.001; ****p < 0.0001. CT, control; DAI, disease activity index; DINF, DSS Infatrini‐Peptisorb®; DIT, DSS Infatrini‐Peptisorb® + TGF‐β addition; DMN, DSS Modulen‐IBD® + TGF‐β neutralization; DSS, dextran sulfate sodium; TGF‐β, transforming growth factor‐β; SEM, standard error of the mean.

**Figure 3:**
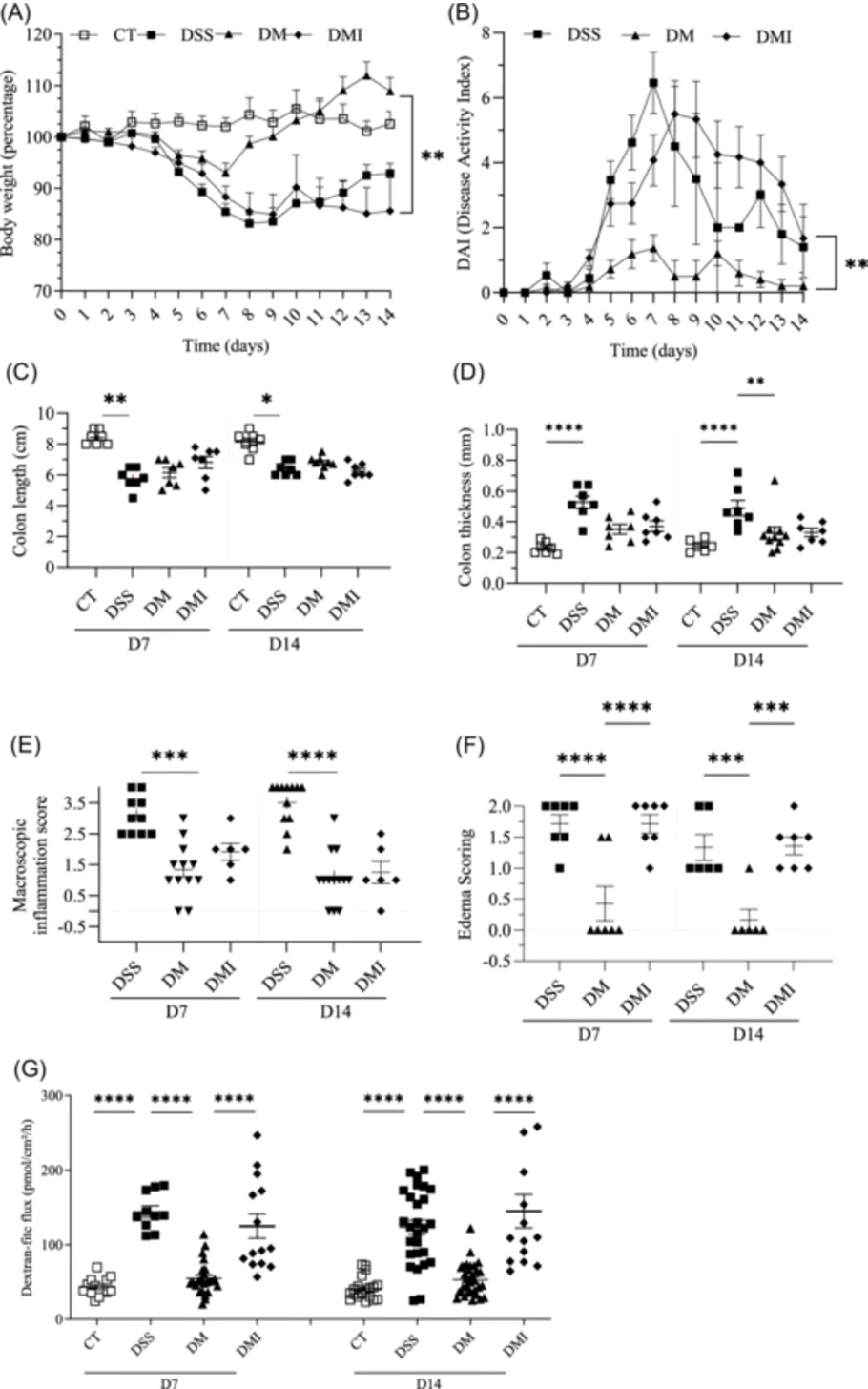
Inhibition of TGF‐β signaling during exclusive enteral nutrition with a TGF‐β rich formula. TGF‐β signaling was inhibited by using SB‐431542 (Selleckchem S1067), antagonist of TGF‐β receptor 1. Inhibition was performed via i.p injections of 5 mg/kg every day from D4 to D7, then every other day from D7 to D14 (DMI). (A) Body weight evolution of CT, DSS, DM (DSS + Modulen‐IBD®), DMI (DSS + Modulen‐IBD® + SB‐431542). (B) DAI during experimentation. (C) Colon length in cm at D7 and D14. (D) Colon thickness in mm at D7 and D14. (E) Macroscopic inflammation score at D7 and D14. (F) Edema scoring at D7 and D14. (G) Intestinal permeability performed in duplicate, each point equivalent to a duplicate. Data are expressed as mean ± SEM; at least *n* > 6 mice per group. *p*‐Values: *p < 0.05; **p < 0.01; ***p < 0.001; ****p < 0.0001. CT, control; DAI, disease activity index; DMI, DSS Modulen‐IBD® + TGF‐β signaling inhibition; DSS, dextran sulfate sodium; TGF‐β, transforming growth factor‐β; SEM, standard error of the mean.

### 3.3 EEN formula containing TGF‐β promotes epithelial cellular properties in 3D organoid model

In mouse fed by EEN, the intestinal restitution promoted by TGF‐β suggests an improvement of epithelial cellular functions. We have then investigated its ability to promote cell survival, proliferation, and differentiation in organoid model, performed at sacrifice D7 or D14, and cultured for up to 9 days. The survival of organoids from DSS crypts was decreased at D7 and D14 (Figure 4A). The mean surface area of mature organoids was higher in DSS condition during culture at the inflammatory peak (D7) compared to those isolated from control mice (Figure 4B). However, at D14, that is, at the recovery period, DSS mature organoids presented a diminished surface area at d3 and d6 of culture, and an increased one at d9 (Figure 4C). The same evolution was observed for immature organoids. This could be explained by a different modulation or delay of enhanced proliferation during the post‐inflammatory remission phase. Moreover, no difference in terms of immature *versus* mature structure number was observed in control, DSS, and DM conditions at D7, and D14 (Figure S5A). Although these DSS structures had an increased surface area during culture, the capacity to generate mature structures (budding i.e., neocrypt) was decreased, with a maximum mean at d9 of culture (Figure 4D). In addition, these DSS organoid bursts due to the excess of apoptotic cells in the lumen were considered as dead organoids (Figure S5B). This shows that cellular properties were impaired in DSS condition. In comparison, DM promoted cell survival, restoring it to control levels (Figure 4A). Immature and mature organoids from crypts of DM‐treated mice had similar growth to control organoids (Figure 4B,C), and had an increased capacity to generate mature structures (budding), as seen at d9 (Figure 4D). The budding structures are comparable to newly formed crypt within the organoid, suggesting a re‐epithelialization capacity.

**Figure 4:**
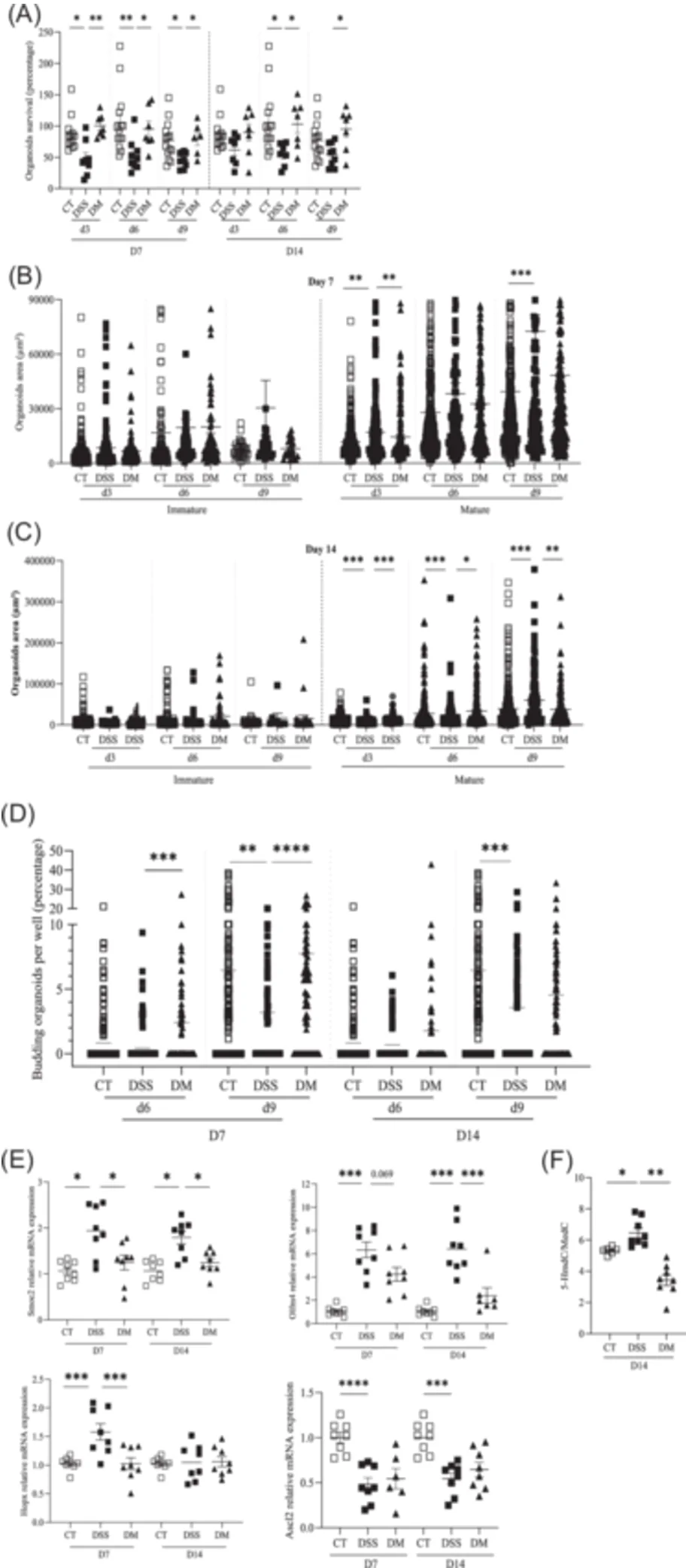
Exclusive enteral nutrition promotes intestinal cellular properties in a 3D organoid model. (A) Organoid survival expressed in percentage from d3 to d9 of culture. (B) Organoids area shown in µm^2^ of immature organoids (left), and mature organoids (right) at the inflammatory peak (sacrifice D7). (C) Organoids area in µm^2^ of immature and mature organoids at the recovery phase (sacrifice D14). (D) Budding organoids per well during culture. Only organoids with at least one bud were counted. (E) mRNA relative expression of *Smoc2, Olfm4, Hopx*, and *Ascl2* from crypts of CT, DSS, and DM mice. (F) Percentage of methylated cytosine at position 5. (F) Data are expressed as mean ± SEM; at least *n* > 6 mice per group. *p*‐Values: *p < 0.05; **p < 0.01; ***p < 0.001; ****p < 0.0001. CT, control; DAI, disease activity index; DSS, dextran sulfate sodium; TGF‐β, transforming growth factor‐β; SEM, standard error of the mean.

In addition to its impact on the ISC abilities to survive, grow and differentiate, DM was able to normalize the mRNA expression of SPARC‐related modular calcium‐binding protein 2 (*Smoc2*), a marker of crypt base columnar (CBC) stem cells at D7, and D14 (Figure 4E). DM was also able to restore a normal mRNA expression of Olfactomedin‐4 (*Olfm4*), a marker of CBC stem cells (Figure 4E). DM also normalized the mRNA expression of Homeodomain‐only protein X (*Hopx*), a marker of +4 stem cells at D7 (Figure 4E). Finally, DSS treatment diminished the mRNA expression of Achaete‐scute complex homolog 2 (*Ascl2*), a marker of CBC, and a normal expression was not restored by DM (Figure 4E). These different cellular properties observed in a physiological *in vitro* environment suggested epigenetic modifications. As expected, the percentage of 5‐methyl deoxycytosine was decreased in colonic epithelium derived from DSS‐treated mice, and normalized in DM conditions (Figure 4F). This suggested that this TGF‐β‐rich formula might restore the methylation at position 5 of cytosine at the CpG sequences, an important epigenetic regulation. However, this TGF‐β‐rich formula was unable to normalize the mRNA expression of colonocyte markers (*Ap1*, *Sis*, and *Fab1*), Goblet cells (*Clca*), enteroendocrine cells (*Cgc*), Tuft cells (*Sucr*), Mucins (*Muc2, 4,* and *3*), antimicrobial peptides (*Tff3* and *Mmp7*), tight junctions (*Ocld*), and receptor of IgA translocation (*PigR*) induced by DSS at D7, and D14 (Figure S6).

## 4 DISCUSSION

Although the beneficial anti‐inflammatory effects of EEN are known, their mechanisms of action remain poorly understood. In our inflammatory model, both EEN formulas promoted weight recovery, and decreased inflammation. However, the presence of TGF‐β in these formulas was required to normalize ISC homeostasis and gut permeability. Thus, our data evidenced the TGF‐β importance to deeply restore intestinal epithelium function.

The TGF‐β‐rich formula improved macroscopic parameters, particularly on weight loss and inflammatory parameters, confirming the results of a previous study.^19^ Other studies also reported the anti‐inflammatory properties of various EEN formulas in IBD mouse models.^5^ Nevertheless, the majority of these studies used EEN formulas that are not specific to CD, and/or presented limits in their experimental protocols. Thus, our study provides a robust murine inflammatory model to address mechanisms of action of a CD‐specific EEN formula. Of note, these beneficial effects were potentially related to several nutritional compounds.^5^ Among them, the casein protein composition induced derivatives such as β‐casofensin, which is known to reduce inflammation.^20^ In addition, aminoacids derived from casein degradation may be involved in the remission process. For example, lysine has anti‐inflammatory properties in a DSS model, and glutamic acid plays an important role in intestinal homeostasis.^21^, ^22^ Similarly, lipids and their derivatives could promote healing effects. Particularly, palmitic acid and medium‐chain triglycerides improve cell proliferation.^23^, ^24^

To better understand the mechanisms of action, we used a second isocaloric formula, Infatrini‐Peptisorb®. Although this formula is not intended for CD, its nutritional composition is quite similar to Modulen‐IBD®. Based on nutritional intake, both formulas restored the body weight of treated mice. Similarly, in Humans, the effect of EEN on clinical remission and inflammatory parameters are induced by several formulas, containing or not TGF‐β.^25^, ^26^ Meanwhile, only the TGF‐β‐rich formula improved macroscopic and edema scores from D7 (inflammatory peak), and normalized intestinal permeability, suggesting a return to an appropriate epithelial functionality. Nevertheless, treatment with the formula that does not contain TGF‐β seemed to improve colonic thickness, and the macroscopic score. This suggests an anti‐inflammatory effect of EEN itself. By comparing the compositions of the two formulas, the quantities of folic acid are higher in Infatrini‐Peptisorb®; folic acid improved the pro‐inflammatory status of BV‐2 microglial cells activated by lipopolysaccharides.^27^ In the 2,4,6‐trinitrobenzene sulfonic acid model, folic acid has been able to maintain Foxp3+ Tregs in the colon, as well as the expression of anti‐apoptotic molecules, Bcl2 and Bcl‐xl, thus preventing colitis.^28^ In humans, folic acid supplementation reduced CRP in various diseases (diabetes, obesity, and metabolic syndrome).^29^ Another difference between these formulas is that Modulen‐IBD® is a polymeric formula, that is, containing proteins in a complete form while Infatrini‐Peptisorb® is a semi‐elemental formula. A study suggested that an elemental formula was less anti‐inflammatory than a polymeric formula in adult mice, while it exacerbated colitis in a DSS model.^30^ However, the nutritional composition of their formulas differs from those we used: 16.7% lipids, 64% carbohydrates, and 19.3% proteins. Finally, data indicated that clinical remission of patients was not affected by the type of formula used.^31^ Therefore, these components do not explain the better results obtained with the naturally TGF‐β‐rich formula.

We subsequently investigated the involvement of TGF‐β in these formulas. The relevance of this cytokine in IBD has been previously demonstrated.^9^, ^32^ In a DSS model, TGF‐β receptor deletion induced exacerbation of colitis.^9^ Furthermore, TGF‐β, present in the extracellular matrix, promoted colonic crypt regeneration.^32^ Concerning TGF‐β levels in patient tissues, one study showed no difference in TGF‐β1 expression between colonic mucosa of IBD patients and healthy subjects.^33^ Similarly, the intestinal mucosa of IBD patients had a defect in the TGF‐β signaling pathway with deregulations of Smads,^8^, ^34^ that is, a decreased phosphorylation of Smad3 in the *lamina propria*, and increased Smad7 in the mucosa and *lamina propria*.^8^, ^35^ Since Smad7 is an inhibitor of the TGF‐β signaling pathway, this blocks its anti‐inflammatory effects. This signaling pathway was re‐established after the inhibition of Smad7.^35^ Thus, the TGF‐β richness of EEN formula could explain the barrier restitution obtained in our model. Our results confirmed those of a previous study carried out on another mouse model of colitis (*IL10* ^−/−^).^36^ Nevertheless, our data are the first to present more detailed results on the components of the intestinal barrier and the ISC capabilities. Moreover, as demonstrated by TGF‐β supplementation experiments, our data highlight the involvement of TGF‐β on colonic thickness, macroscopic and edema scores, as well as normalization of colonic permeability. These effects on macroscopic and microscopic parameters demonstrate that TGF‐β potentiates EEN effects. Moreover, considering the loss of these effects after TGF‐β neutralization, we demonstrated the importance of this cytokine in mucosal healing and functionality during EEN. This phenomenon was notably observed in a clinical study, where only patients treated with Modulen‐IBD® had an improvement on histological inflammation.^37^ Also, supplementation with TGF‐β (DIT) showed a similar level of resolution as the natural presence of TGF‐β (DM), notably with the decrease of edema and normalization of intestinal permeability. Moreover, the effects obtained with TGF‐β were more accentuated at D14, that is, at the recovery phase, suggesting the importance of this cytokine for cellular regeneration.

Finally, TGF‐β is essential to restore a functional epithelium, assessed by mucosal healing with normalization of intestinal permeability. Therefore, we studied the intestinal renewal using the organoid model, by comparing the survival, growth, and ability of ISC to proliferate and differentiate into a functional epithelium, coming from colonic mice treated by a TGF‐β‐rich formula (DM). The organoid model was used to study the gut epithelium and its capacities independently of the other components of the intestinal barrier. We found that organoid survival was decreased under inflammatory conditions, reflecting the inability of DSS‐treated crypts to close and form organoids. Similarly, these organoids showed increased proliferation, which may be caused by the alteration of cellular pathways of proliferation and differentiation, demonstrated also by the decrease in neo‐crypt formation. These parameters were improved in organoids developed from DM mice. In particular, the formation of neo‐crypts was increased, suggesting an enhanced capacity of re‐epithelialization of these cells, thus an increased ability to induce mucosal functional healing.

The organoid model enables also to study genomic imprinting of ISCs, described to participate in controlling their survival, growth, and differentiation. Thus, the experimental models of colitis were described to alter the ISC ability to survive, grow, and differentiate into a mature epithelium as well as to modify the genomic imprinting of ISC.^38^ Moreover, DNA methylation was also described to be altered in IBD patients, and appeared to be involved in their pathophysiology.^39^ In agreement with these data, our results evidenced that experimental colitis induced by DSS altered ISC ability to survive, grow, and differentiate into a mature epithelium as well as modified genomic imprinting by increasing DNA methylation. In addition, our data reported that colitis was able to affect the mRNA expression of several markers of CBC (*Ascl2, Olfm4*, and *Smoc2*), and +4 ISC (*Hopx*). Finally, our results have shown that the formula containing TGF‐β was effective to normalize ISC ability to survive, grow, and differentiate into a mature epithelium, to reduce DNA methylation as well as to restore mRNA expression of ISC markers. Epigenetics deserves to be studied in murine and clinical studies to better understand the mechanisms of EEN, as shown by our results that highlight for the first time the possible epigenetic modifications induced by EEN, including the naturally TGF‐β‐rich formula, in IBD.

## 5 CONCLUSION

This study demonstrates the anti‐inflammatory and pro‐regenerative properties of EEN treatment in IBD. Although EEN appears to be involved in the improvement of colitis whatever the formulas are, TGF‐β plays a major role in restoring the barrier function and the ISC function. Finally, cellular pro‐regenerative capacities are improved, probably through genomic imprinting. Our results are the first to provide details on the mechanisms by which EEN promotes the epithelium restitution in a mouse model of CD. Future studies should explore which signaling pathways involved in both proliferative and differentiation processes are stimulated by TGF‐β in CD to renew a functional epithelium. It could enable to identify new therapeutic targets, and to improve the compliance of patients with CD.

## CONFLICT OF INTEREST STATEMENT

The authors declare no conflicts of interest.

## Supporting information


**Supplementary Figure 1**. Detection of TGF‐β by ELISA in exclusive enteral nutrition formulas. TGF‐β2 in EEN formulas was quantified by ELISA (Cusabio CSB‐E14209B). CurveExpert software was used for the curve fit. Data are expressed as mean ± SEM;
**Supplementary Figure 2**. Exclusive enteral nutrition induces clinical remission in pediatric CD patients. Twelve children with CD treated by exclusive enteral nutrition (EEN) with Modulen IBD® at Toulouse Children Hospital. Clinical research project BIODIGE: NCT02874365. A: Body weight evolution during treatment. B: CRP levels before and at day 15 of exclusive enteral nutrition (EEN). C: Albumin level. There is an improvement of inflammation (B), and protein loss (C) by EEN, before introduction of immunosuppressive drugs or biologics. Data represented as mean ± SEM. *P‐values*: **p < 0.01;
**Supplementary Figure 3**. Exclusive enteral nutrition alleviates microscopic inflammation. A: Hematoxylin and eosin staining of control (A), DSS (B), and DM (C) colon sections. B: Histological score at day 7 (D7) and D14 of DSS and DM colon rolls (two samples per each);
**Supplementary Figure 4**. Exclusive enteral nutrition induces remission in *Nod2* ^*KO*^ mice. A: Body weight monitoring of CT, DSS, and DM treated *Nod2* ^*KO*^ mice (legend at the top). B: Disease activity index (DAI) during DSS procedure. C: Colon length at day 7 (D7) and D14 expressed in cm. D: Colon thickness at D7 and D14 expressed in mm. E: Macroscopic inflammatory score at D7 and D14. F: Dextran‐FITC flux representing intestinal permeability performed in duplicate, each point equivalent to a duplicate. Results are expressed as mean ± SEM; n = 7 mice per group. *P‐values*: *p < 0.05; **p < 0.01; ***p < 0.001; ****p < 0.0001 vs. DSS group;
**Supplementary Figure 5**. Organoid structure analysis. A: Percentage of immature and mature organoids among counted organoids during culture. B: Funnel chart of dead organoid percentage in each condition during culture (top, middle of each funnel bloc). Percentage of dead organoids at day 6 or day 9, considering the maturity category at the previous analysis day (e.g: among 47% of DSS dead organoids at d6 of culture, 49% of them were immature at d3 of culture);
**Supplementary Figure 6**: Quantitative PCR of CT, DSS, DM crypts. Relative mRNA expressions of markers of colonocytes (A: *Ap1*, B: *Sis*, C: *Fabp1*), enteroendocrine cells (D: *Cgc*), Goblet cells (E: *Clca*), Tuft cells (F: *Sucr*), Mucins (G: *Muc2*, H: *Muc3*, I: *Muc4*), antimicrobial peptides (J: *Tff3*, K: *Mmp7*), tight junctions (L: *Ocld*), and receptor of IgA translocation (M: *PigR*). Data are expressed as mean ± SEM; At least n > 4 mice per group. Statistics: CT *vs*. DSS, and DM *vs*. DSS. *P‐values*: * p < 0.05; ** p < 0.01; ***p < 0.001; ****p < 0.0001;
**Supplementary Table 1**: Gene sequences used for qPCR. NCBI design tool was used to design sequences;
**Supplementary Table 2**: Nutritional composition of Modulen IBD® and Infatrini Peptisorb® per 100 ml of liquid. The percentage of differences was calculated with Modulen IBD® as reference.

SF1.

SF2.

SF3.

SF4.

SF5.

SF6.

ST1.

ST2.

## Data Availability

Relevant information about data are available directly from the corresponding author.
