# Supplementary material for: Exclusive enteral nutrition containing transforming growth factor‐β improves intestinal barrier function in a colitis mouse model
Source: J Pediatr Gastroenterol Nutr. 2026 Jul 21;83(4):692–702. doi: 10.1002/jpn3.70517 (PMC13646034; doi:10.1002/jpn3.70517)
Supplement: Supplementary file 1 [file JPN3-83-692-s001.pdf]

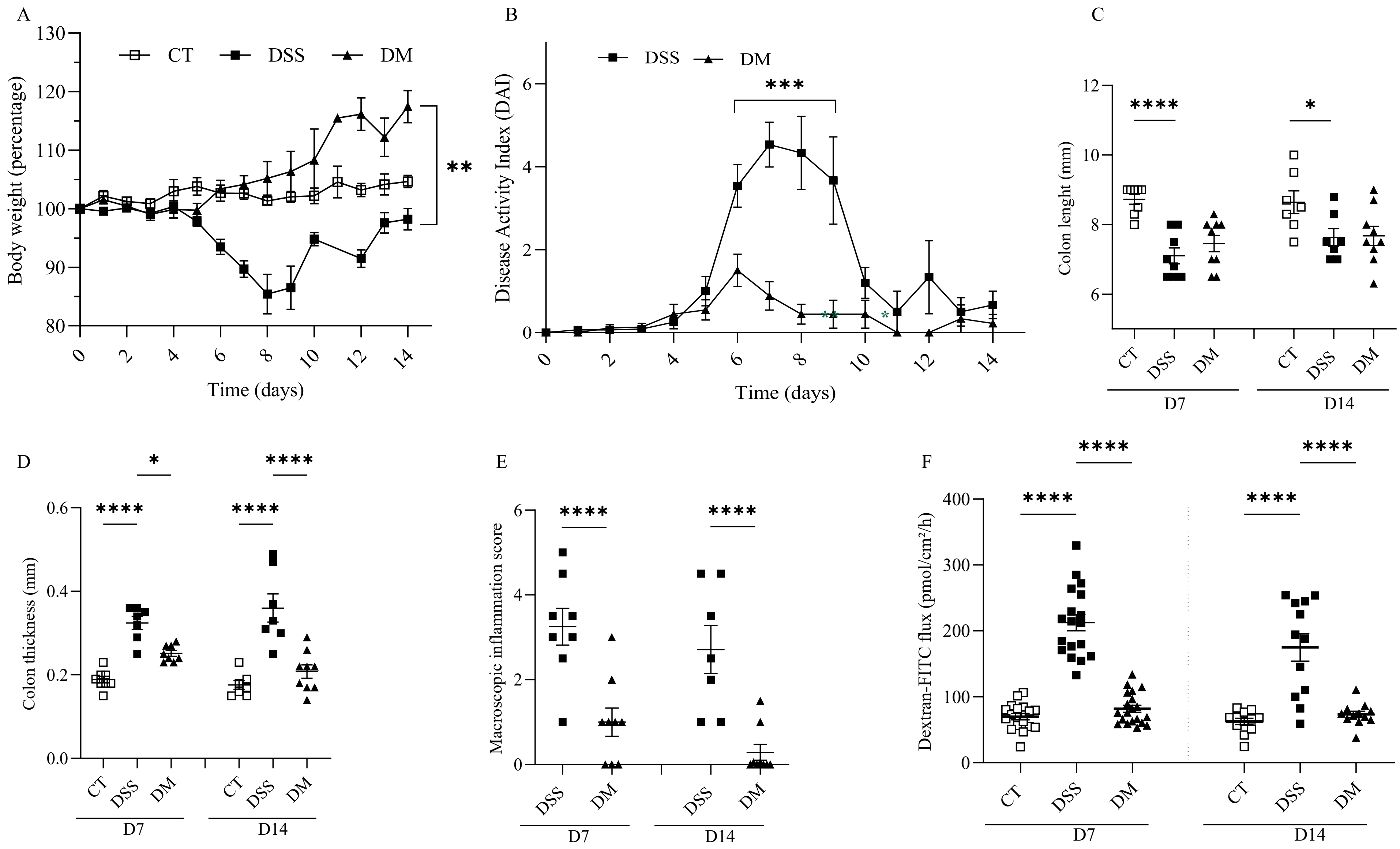

A
CT
DSS
DM
Body weight (percentage)
130
120
110
100
90
80
**
Time (days)
0
2
4
6
8
10
12
14
B
DSS
DM
***
Disease Activity Index (DAI)
6
4
2
0
Time (days)
C
Colon lenght (mm)
12
10
8
6
****
*
CT
DSS
DM
D7
D14
D
Colon thickness (mm)
0.6
0.4
0.2
0.0
****
*
****
****
E
Macroscopic inflammation score
****
****
F
Dextran-FITC flux (pmol/cm²/h)
400
300
200
100
0
****
****
****
****
