# Supplementary material for: Exclusive enteral nutrition containing transforming growth factor‐β improves intestinal barrier function in a colitis mouse model
Source: J Pediatr Gastroenterol Nutr. 2026 Jul 21;83(4):692–702. doi: 10.1002/jpn3.70517 (PMC13646034; doi:10.1002/jpn3.70517)
Supplement: Supplementary file 2 [file JPN3-83-692-s003.pdf]

	Per 100 ml (1.0 Kcal/ml)		
	Modulen IBD®	Infatrini Peptisorb®	%
**Energy (Kcal)**	98.6	100	101
**Carbohydrates (g)**	11	10.2	93
**Proteins (g)**	3.5	2.6	74
**Fats (g)**	4.6	5.4	117
**SFAs (g)**	2.6	3.8	146
**MCTs (g)**	1.2	2.8	233
**MFAs (g)**	0.78	0.77	99
**PFAs (g)**	0.50	0.86	172
**α linolenic acid (mg)**	40	70	175
**Linoleic acid (mg)**	420	690	164
**Vitamins**			
**A (µg)**	82	88	107
**D (µg)**	0.98	2.4	245
**E (mg)**	1.3	2.1	162
**K (µg)**	5.4	6.7	124
**C (mg)**	9.4	14	149
**Thiamin (mg)**	0.12	0.15	125
**Riboflavin (mg)**	0.13	0.2	154
**Niacin (mg)**	1.2	0.8	67
**B6 (mg)**	0.17	0.11	65
**Folic acid (µg)**	24	16	67
**B12 (µg)**	0.32	0.3	94
**Biotin (µg)**	3.2	4	125
**PA (mg)**	0.48	0.8	167

SFAs : Saturated Fatty acids; MCTs: Medium; chain triglycerides; MFAs: Monounsaturated fatty acids; PFAs : Polyunsaturated fatty acids; PA: Pantothenic acid

	Per 100 ml (1.0 Kcal/ml)		
	Modulen IBD®	Infatrini Peptisorb®	%
**Sodium (mg)**	34	37	109
**Potassium (mg)**	120	111	93
**Chloride (mg)**	73	75	103
**Calcium (mg)**	89	90	101
**Phosphorus (mg)**	60	45	75
**Magnesium (mg)**	20	9	45
**Iron (mg)**	1.1	1,2	109
**Zinc (mg)**	0.94	0.8	85
**Copper (mg)**	0.098	0.075	77
**Manganese (mg)**	0.20	0.006	3
**Fluoride (mg)**		-	
**Selenium (µg)**	3.4	3.75	110
**Chromium (µg)**	5	4	80
**Molybdenum (µg)**	7.4	6	81
**Iodine (µg)**	9.8	19	194
**Other nutrients**			
**Choline (mg)**	7	32.1	459
**Carnitine (mg)**	-	2	
**Taurine (mg)**	-	7	
**Inositol (mg)**	-	25	
**Osmolarity (mOsm/L)**	290	295	102
