# Supplementary material for: Exclusive enteral nutrition containing transforming growth factor‐β improves intestinal barrier function in a colitis mouse model
Source: J Pediatr Gastroenterol Nutr. 2026 Jul 21;83(4):692–702. doi: 10.1002/jpn3.70517 (PMC13646034; doi:10.1002/jpn3.70517)
Supplement: Supplementary file 3 [file JPN3-83-692-s004.pdf]

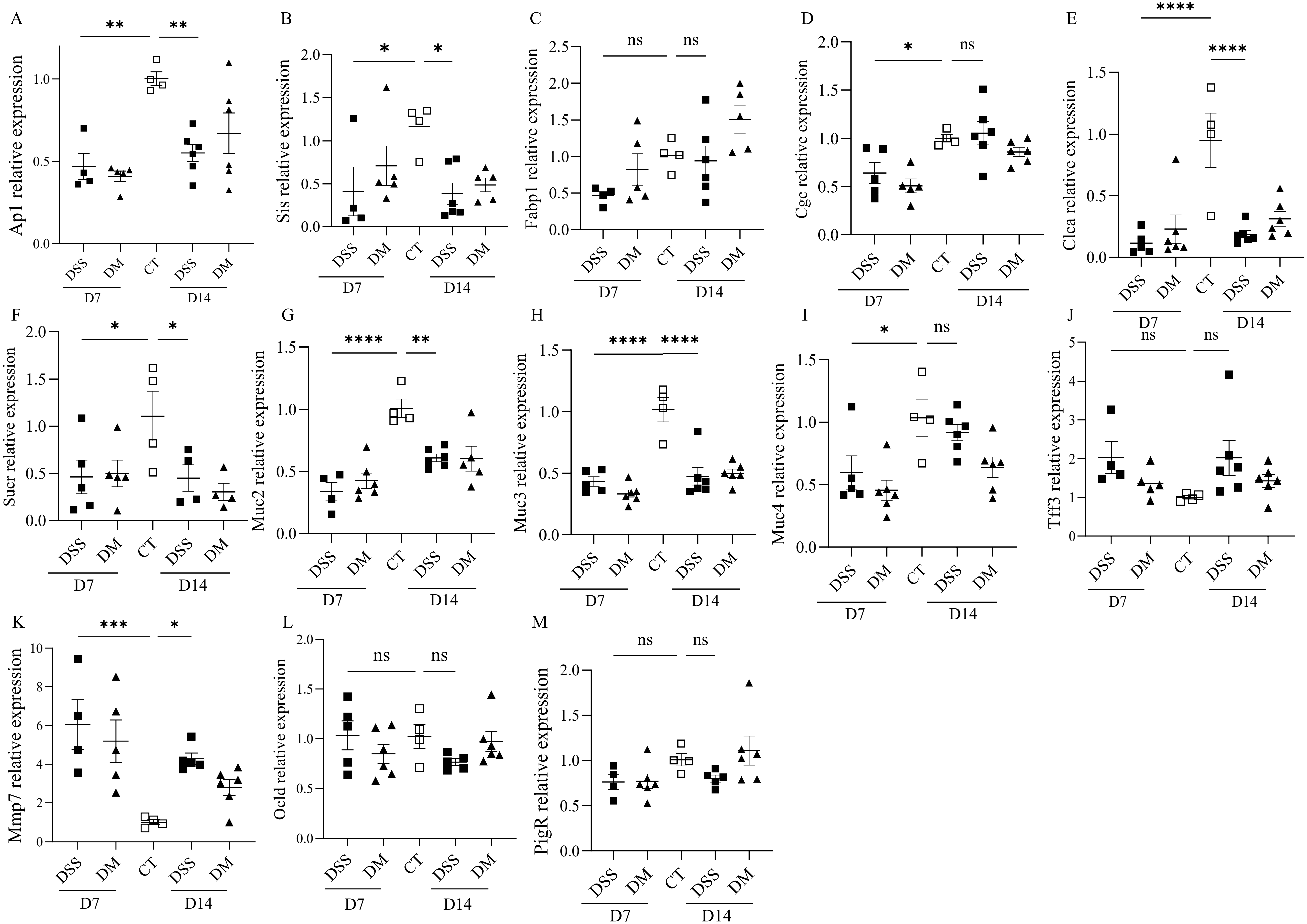
A
Ap1 relative expression
**
**
B
Sis relative expression
*
*
C
Fabp1 relative expression
ns
ns
D
Cgc relative expression
*
ns
E
Clca relative expression
****
****
F
Sucr relative expression
*
*
G
Muc2 relative expression
****
**
H
Muc3 relative expression
****
****
I
Muc4 relative expression
*
ns
J
Tff3 relative expression
ns
ns
K
Mmp7 relative expression
***
*
L
Ocld relative expression
ns
ns
M
PigR relative expression
ns
ns
DSS
DM
CT
DSS
DM
D7
D14
