# Supplementary material for: Exclusive enteral nutrition containing transforming growth factor‐β improves intestinal barrier function in a colitis mouse model
Source: J Pediatr Gastroenterol Nutr. 2026 Jul 21;83(4):692–702. doi: 10.1002/jpn3.70517 (PMC13646034; doi:10.1002/jpn3.70517)
Supplement: Supplementary file 4 [file JPN3-83-692-s005.pdf]

A

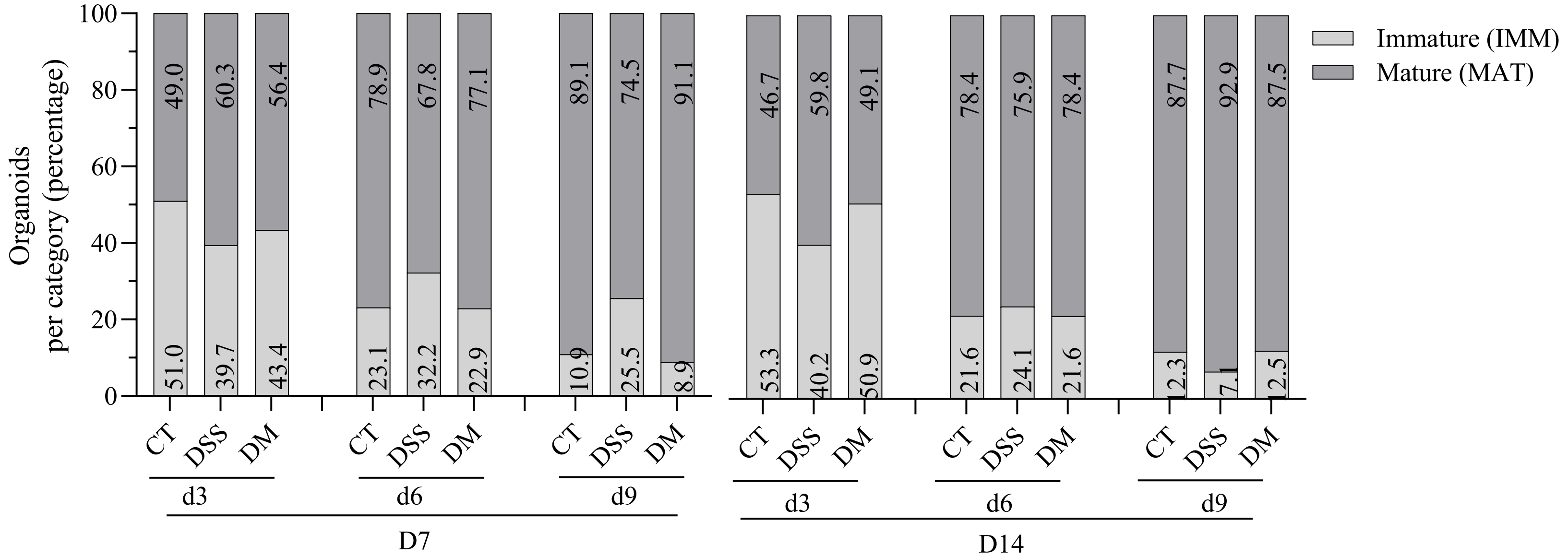

Organoids per category (percentage)
100
80
60
40
20
0
Immature (IMM)
Mature (MAT)
49.0
60.3
56.4
51.0
39.7
43.4
78.9
67.8
77.1
23.1
32.2
22.9
89.1
74.5
91.1
10.9
25.5
8.9
46.7
59.8
49.1
53.3
40.2
50.9
78.4
75.9
78.4
21.6
24.1
21.6
87.7
92.9
87.5
12.3
7.1
12.5
CT
DSS
DM
d3
d6
d9
D7
D14

B

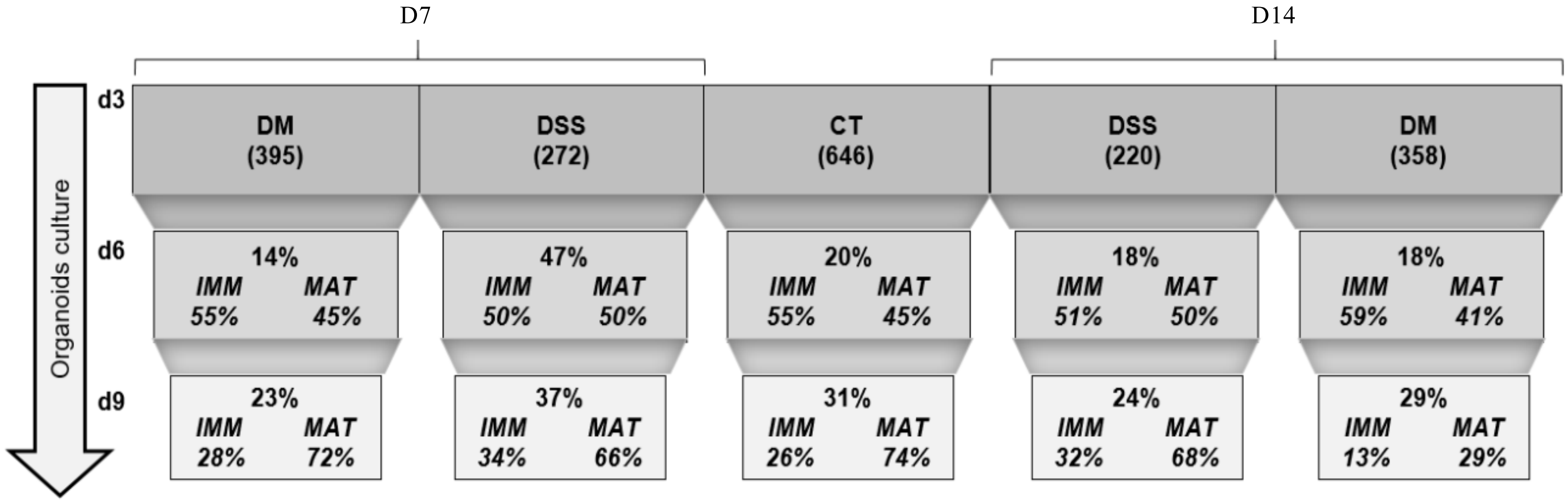

D7
D14
Organoids culture
d3
d6
d9
DM (395)
DSS (272)
CT (646)
DSS (220)
DM (358)
14%
IMM 55%
MAT 45%
47%
IMM 50%
MAT 50%
20%
IMM 55%
MAT 45%
18%
IMM 51%
MAT 50%
18%
IMM 59%
MAT 41%
23%
IMM 28%
MAT 72%
37%
IMM 34%
MAT 66%
31%
IMM 26%
MAT 74%
24%
IMM 32%
MAT 68%
29%
IMM 13%
MAT 29%
