# Supplementary material for: Exclusive enteral nutrition containing transforming growth factor‐β improves intestinal barrier function in a colitis mouse model
Source: J Pediatr Gastroenterol Nutr. 2026 Jul 21;83(4):692–702. doi: 10.1002/jpn3.70517 (PMC13646034; doi:10.1002/jpn3.70517)
Supplement: Supplementary file 5 [file JPN3-83-692-s006.pdf]

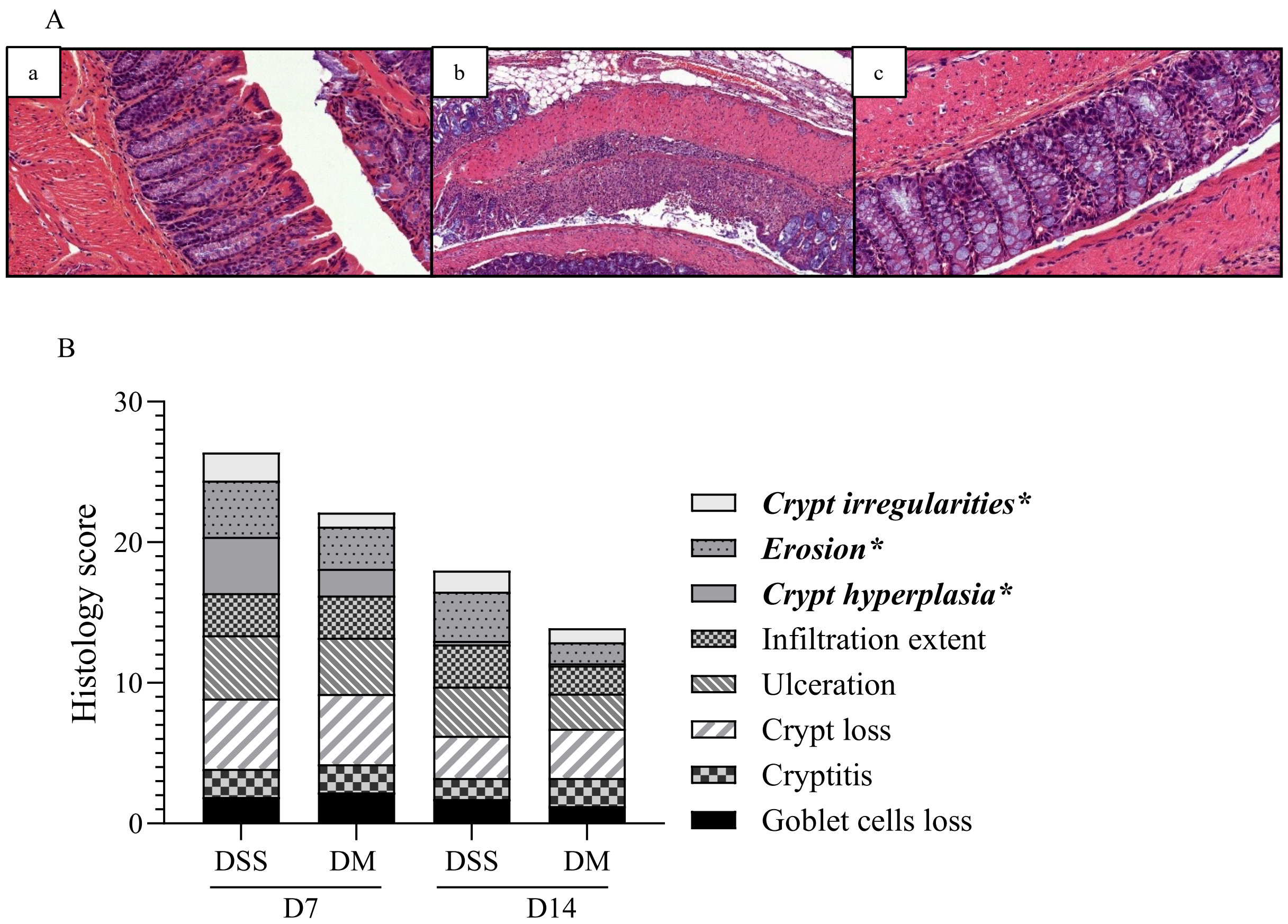

A
a
b
c
B
Histology score
30
20
10
0
DSS
DM
DSS
DM
D7
D14
Crypt irregularities*
Erosion*
Crypt hyperplasia*
Infiltration extent
Ulceration
Crypt loss
Cryptitis
Goblet cells loss
