# Supplementary material for: Exclusive enteral nutrition containing transforming growth factor‐β improves intestinal barrier function in a colitis mouse model
Source: J Pediatr Gastroenterol Nutr. 2026 Jul 21;83(4):692–702. doi: 10.1002/jpn3.70517 (PMC13646034; doi:10.1002/jpn3.70517)
Supplement: Supplementary file 6 [file JPN3-83-692-s007.pdf]

Name	Forward sequence	Reverse sequence
Ap1	GGGAAGCGTGTTCTGGCTAT	GCACATCACCACTACACCGA
Achaete-scute family bHLH transcription factor 2 (*Ascl2*)	GCCTACTCGTCGGAGGAA	CCAACTGGAAAAGTCAAGCA
Cgc	TACACCTGTTCGCAGCTCAG	GCTGGGAATGATCTGGGGTT
Clca	GCCGGAAGATGAAGCCCTC	TTGATACAAGGACATCAGCGTTTTT
Cxcl8	AGCCACACTCAAGAATGGTC	GTCAGAAGCCAGCGTTCAC
Fatty acid binding protein 1 (*Fabp1*)	GGGGAAAAAGTCAAGGCAGTC	TGTCGCCCAATGTCATGGT
Gapdh	AGGTCGGTGTGAACGGATTTG	TGTAGACCATGTAGTTGAGGTCA
Hopx	CATCTATCTCGCCCCCGTC	CAGGCGCTGCTTAAACCATT
MMP7	AGCTCTGAGAACAATCGCCTG	GCAGCAGACACACAAAGCAG
Mucin 2 (*Muc2*)	TGACTGCCGAGACTCCTACA	CTGTAGTGTGGGGTGCTGAC
Mucin 3 (*Muc3*)	CTCTGCCTGCTGGCTTTCAT	CTGTTTTCCCCGTCTGTGGTT
Mucin 4 (*Muc4*)	TGGCTACAAAGGCTACCACC	CCCTCACTACATGGGGACAC
Occludin	TTCTCCCGCAACTGGCATC	GAGTGAAGAGTACATGGCTGCT
Olfm4	CAGCTGCCTGGTTGCCTCCG	GGCAGGTCCCATGGCTGTCC
Pigr	TGGTGTGGGGTAAAGCAAGG	GACATGGGATGACCCTCTGG
Sis	GTGCCTGCTGTGGAAGAAGTA	AACAGCCACGCTCTTCACAT
SPARC-related modular calcium-binding protein 2 (*Smoc2*)	GCAGGGAAAGCAGATGATGC	CTCGGTCCAGAGTGTAGGGT
Sucrase-isomaltase (*Si*)	GTGCCTGCTGTGGAAGAAGTA	TGTCGCCCAATGTCATGGT
TFF3	GGAGCCTGGACAGCTTCAAA	ACGTTGGCCTGTCTCCAAG
