# Supplementary material for: Exclusive enteral nutrition containing transforming growth factor‐β improves intestinal barrier function in a colitis mouse model
Source: J Pediatr Gastroenterol Nutr. 2026 Jul 21;83(4):692–702. doi: 10.1002/jpn3.70517 (PMC13646034; doi:10.1002/jpn3.70517)
Supplement: Supplementary file 7 [file JPN3-83-692-s008.pdf]

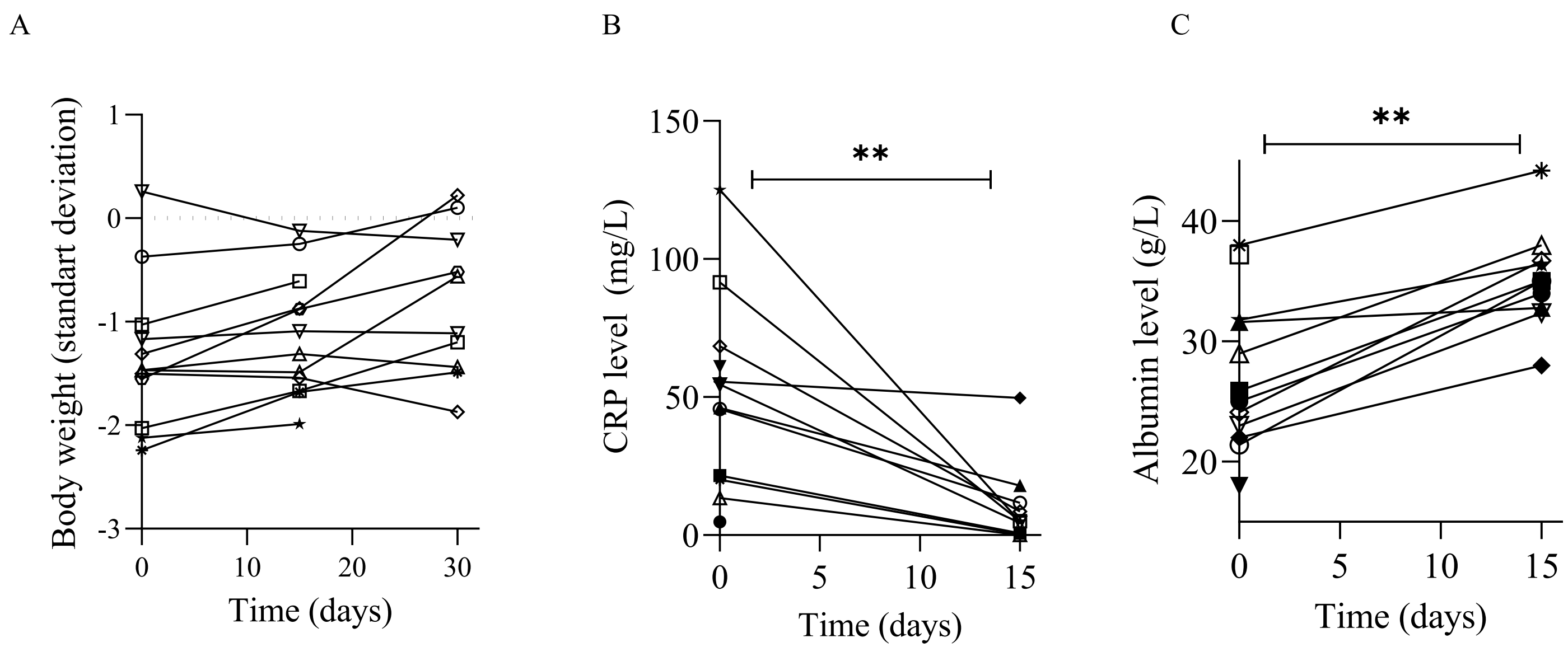

A
Body weight (standart deviation)
1
0
-1
-2
-3
0
10
20
30
Time (days)
B
CRP level (mg/L)
150
100
50
0
0
5
10
15
Time (days)
**
C
Albumin level (g/L)
40
30
20
0
5
10
15
Time (days)
**
