# Supplementary material for: Exclusive enteral nutrition containing transforming growth factor‐β improves intestinal barrier function in a colitis mouse model
Source: J Pediatr Gastroenterol Nutr. 2026 Jul 21;83(4):692–702. doi: 10.1002/jpn3.70517 (PMC13646034; doi:10.1002/jpn3.70517)
Supplement: Supplementary file 8 [file JPN3-83-692-s009.pdf]

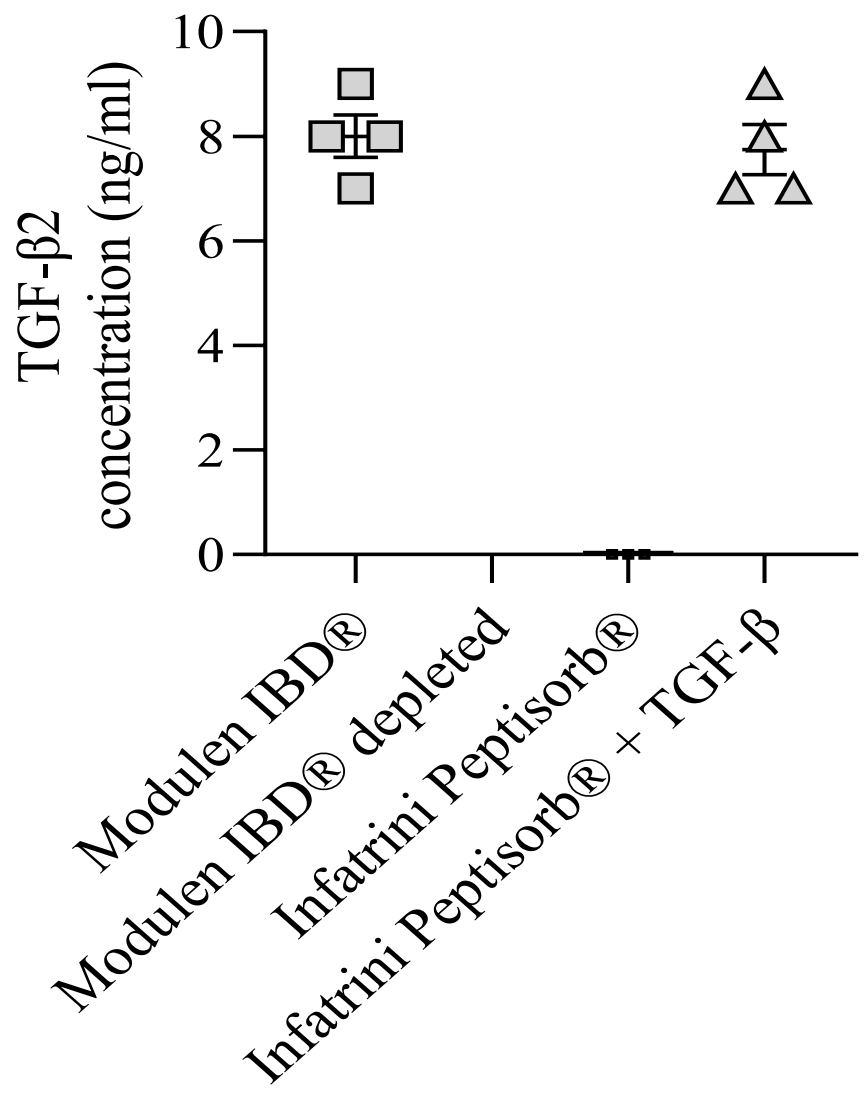
TGF-β2
concentration (ng/ml)
10
8
6
4
2
0
Modulen IBD®
Modulen IBD® depleted
Infatrini Peptisorb®
Infatrini Peptisorb® + TGF-β
