# Supplementary material for: Exclusive enteral nutrition containing transforming growth factor‐β improves intestinal barrier function in a colitis mouse model
Source: J Pediatr Gastroenterol Nutr. 2026 Jul 21;83(4):692–702. doi: 10.1002/jpn3.70517 (PMC13646034; doi:10.1002/jpn3.70517)
Supplement: Supplementary file 9 [file JPN3-83-692-s002.docx]

**Supplementary material and methods**

**Experimental procedures.** Mice were treated with dextran sulfate sodium (DSS) at 3% during 5 days to induce a colitis, and then DSS was replaced by regular water (DSS group). Progression of the disease was monitored daily until D14. Instead of regular food, other mice received EEN from day D5 to D7 or D14. We used two formulas *ad libitum*, a naturally TGF-β-rich formula (Modulen-IBD® (*Nestlé*)) (DM group) and another formula that does not contain TGF-β (Infatrini-Peptisorb® (*Nutricia*)) (DINF group). For TGF-β neutralization, 1D11 antibody *(ThermoFischer Scientific)* was added to Modulen-IBD® formula during the treatment (DMN group). For TGF-β signaling inhibition, SB431542 *(Selleckchem)* was diluted in DMSO, PEG300 and distilled H2O following the manufacturer’s instructions, and administrated *via* *i.p* every day from D4 to D7, then every other day from D7 to D14 (DMI group). Finally, recombinant TGF-β2 protein expressed in mammalian cell (*MBS954812)* was added to Infatrini-Peptisorb® at 10 ng/mL and administrated to mice *ad libitum* (DIT) group. TGF-β concentrations were measured by ELISA *(Cusabio)* according to their protocol.

**Clinical observations.** To assess colitis severity, the body weight of mice was monitored daily in order to establish the disease activity index (DAI) adapted from Van der Slui et al.(1) We attributed points for weight loss as following: 1 point if weight loss was between >5% and <10%, 2 points if ⩾10% and <15%, 3 points if ⩾15% and <20%, and 4 points if ⩾20%. Presence of blood in stools or around anus corresponded to 1 point. Non-formed stools corresponded to 1 point, mild diarrhea to 2 points, and severe diarrhea to 3 points. Physical appearance was also observed: 1 point for bristling fur, 2 points if hunched back, 3 points if both, and 4 points if the mice was lethargic.

**Macroscopic analysis.** A macroscopic inflammation score was determined by analyzing colon appearance, taking into account edema length (1 point if <1 cm; 1.5 if ⩾1 cm and < 2cm; 2 points if ⩾2cm), and colon wall thickness measuring with a caliper (0 points if <0.3mm, 1 point if ⩾0.3 and < 0.45mm, 1.5 points if ⩾0.45 and <0.55mm, and 2 points if ⩾0.55mm). Colon length (cm) was also monitored.

**Histological analysis.** To establish histological analysis, colon rolls were established. The colon was enrolled, fixed in formol overnight, then in 70% ethanol at 4°C. After inclusion in paraffin, sections of 5 µm were made for hematoxylin/eosin coloration. Photos were acquired with Panoramic Scan Flash III, and analyzed with CaseViewer. A minimum of 50 crypts by sample, from the entire sample, was measured, and their cell number was counted. For each condition, two different samples were analyzed. The histological score was adapted from the literature,(2) and points were attributed as: inflammatory cell infiltrate extend (Mucosal: 1, Mucosal and submucosal: 2, Mucosal, submucosal and transmural: 3), epithelial changes (Hyperplasia (minimal <25%: 1, Mild 25-35%: 2, Moderate 36-50%: 3, Marked > 51%: 4), Goblet cell loss (minimal <20%: 1, Mild 21-35%: 2, Moderate 36-50%: 3, Marked > 50%: 4), Cryptitis: 2 to 3, Crypt abscesses: 3 to 5, Erosion: 1 to 4), mucosal architecture (Ulceration : 3 to 5, Irregular crypts: 4 to 5, Crypt loss: 4 to 5).

**Colonic organoid culture**. After sacrifice, colons were opened longitudinally and washed in PBS without calcium and magnesium, then transferred to a dissociation medium (PBS, EDTA 9 mM, DTT 3 mM, Y27632 10 µM). The crypts’ dissociation was conducted with an orbital spinning wheel for 40 minutes at room temperature followed by a mechanical re-suspension.(3-5) After discarding the colon residues, the tube contents were centrifuged at 16g for 5 min at 4° C. The pellet containing the isolated intestinal crypts was washed with 3 ml of DMEM/F12 medium, and 60 crypts were embedded in 10µl supplemented hEs-qualified matrix per well (matrigel, 100 ng/ml murin noggin, 50 ng/ml murin EGF, 1 µM Jagged, 1.3 µM CHIR). The growth medium was added after a matrigel polymerization time of 15 min at 37°C and was changed every three days (Wnt3a medium 50%, advanced DMEM/F12 50%, glutamax 2 mM, hepes 1 mM, 1X B27, 100 U/ml penicillin/streptomycin, 1X N2, 200 ng/ml R-spondin, 0.5 mM N-acetylcystein, 10µM Y27632, 100 ng/ml murin noggin, 50 ng/ml murin EGF). The crypts of each colon were plated in 15 wells µ-Slide Angiogenesis Ibidi® plate.(5) Organoids were maintained in a humidified incubator at 37°C under 5% CO_2_ and 95% air atmosphere. Organoid stem cell survival (number of organoids formed), and growth capacity (organoids area (µm²)) were followed three, six, and nine days after plating with a wide field transmission microscope (Apotome Zeiss, 10X lens). The ImageJ® software was used to process and measure the organoid size, and survival of crypts, and buds were quantified by counting the number of organoids and buds formed in each well as previously described.(4, 5)

**RNA extraction, reverse transcription and quantitative PCR.** RNA from colonic crypts was extracted by Trizol reagent.(6) Briefly, 1 ml of Trizol was added to each sample, followed by 200 µl of chloroform. The aqueous phase obtained after centrifugation was mixed with isopropanol. After pellet discard, ethanol was used to wash the RNAs. Purification of RNAs from DSS was done as described in the literature.(7) The RNA concentrations were determined by Nanodrop (Nanophotometer Implen P330). Acceptable absorbance ratio at 260/230 nm was over 2, whereas acceptable ratio at 260/280 nm was from 1.8 to 2.2. The reverse transcription was done with M-MLV according to the manufacturer’s protocol, and PCR reaction was performed using a LightCycler® 480 Real-time PCR system device using Takyon with the primer sequences detailed in Supplementary Table 1.

**DNA methylation.** The levels of 5-methyl deoxycytosine (5-Me-dC) were measured by high-performance liquid chromatography–mass spectrometry(HPLC-MS/MS) as previously described.(4) DNA from frozen cell pellets (−80°C) was extracted using the DNeasy® Blood and Tissue kit (*Qiagen*) according to the manufacturer’s procedure. A RNase treatment step was added after cell lysis; samples were incubated for 2 min with 400 μg of RNaseA. DNA was eluted in 100 μl of 0.1 mM deferoxamine to prevent spurious oxidation. DNA from 50 μl of the sample was digested in two incubation steps. First, the pH was adjusted to pH 5.5 by adding 5 μl of buffer (100 mM succinic acid, 50 mM calcium chloride, 150 mM, 5 μM zinc sulfate, pH 5.5). Samples were then incubated for 2h at 37°C in the presence of 2.5 U of nuclease P1, DNase II, and 0.05 U of phosphodiesterase II. Then, 6 μL of Tris buffer (500 mM Tris, 1 mM ethylenediaminetetraacetic acid, pH 8) was added with 2 U of alkaline phosphatase and 0.05 U of phosphodiesterase I. The sample was incubated for a further 2h at 37°C. These samples were neutralized with HCl 0.1 N, filtered on 0.22 µm filter units to eliminate any remaining particles, and injected onto the HPLC/MS-MS. The spectrometer was used in the MRM3 mode with positive electrospray ionization. The monitored fragmentation was m/z 284 [M+H]+ → 168 [M+H -2-deoxyribose]+ →140 [M+H -2-deoxyribose−CO]+. Chromatographic separations were achieved using a C18 reversed-phase Uptisphere ODB column (*Interchim, Montluçon, France*). The elution was performed using a gradient of acetonitrile in 2mM ammonium formate, at a flow rate of 0.2 ml/min. The retention time was 20 min. In addition to the MS spectrometer, the HPLC eluent was analyzed in a UV detector set at 270 nm to quantify the number of unmodified nucleosides.

**References**

1	Van der Sluis M, De Koning BA, De Bruijn AC, et al. Muc2-deficient mice spontaneously develop colitis, indicating that MUC2 is critical for colonic protection. Gastroenterology 2006;131:117–29.

2	Erben U, Loddenkemper C, Doerfel K, et al. A guide to histomorphological evaluation of intestinal inflammation in mouse models. Int J Clin Exp Pathol 2014;7:4557–76.

3	Laborde N, Barusseaud A, Quaranta M, et al. Human colonic organoids for understanding early events of familial adenomatous polyposis pathogenesis. J Pathol 2025;265:26–40.

4	Carle C, Boucher D, Morelli L, et al. Perinatal foodborne titanium dioxide exposure-mediated dysbiosis predisposes mice to develop colitis through life. Part Fibre Toxicol 2023;20:45.

5	Sola Tapias N, Denadai-Souza A, Rolland-Fourcade C, et al. Colitis Linked to Endoplasmic Reticulum Stress Induces Trypsin Activity Affecting Epithelial Functions. J Crohns Colitis 2021;15:1528–41.

6	Alnabhani Z, Montcuquet N, Biaggini K, et al. Pseudomonas fluorescens alters the intestinal barrier function by modulating IL-1beta expression through hematopoietic NOD2 signaling. Inflamm Bowel Dis 2015;21:543–55.

7	Viennois E, Tahsin A, Merlin D Purification of Total RNA from DSS-treated Murine Tissue via Lithium Chloride Precipitation. Bio Protoc 2018;8:e2829.

**Supplementary figures legends.**

**Supplementary Figure 1. Exclusive enteral nutrition alleviates microscopic inflammation.**

**A:** Hematoxylin and eosin staining of control (A), DSS (B), and DM (C) colon sections. **B:** Histological score at day 7 (D7) and day 14 (D14) of DSS and DM colon rolls (two samples per each).

**Supplementary Figure 2. Exclusive enteral nutrition induces remission in *Nod2^KO^* mice.**

**A:** Body weight monitoring of CT, DSS and DM treated *Nod2^KO^* mice (legend at the top). **B:** DAI (disease activity index) during DSS procedure, and DM *Nod2^KO^* mice (same legend as A). **C:** Colon length at day 7 (D7) and day 14 (D14) expressed in cm. **D:** colon thickness at D7 and D14 expressed in mm. **E**: macroscopic inflammatory score at D7 and D14. **F:** Dextran-FITC flux representing intestinal permeability performed in duplicate, each point equivalent to a duplicate. Results are expressed as mean ± SEM; at least n>7 mice per group. *P-values*: * p<0.05; ** p<0.01; ***p<0.001; ****p<0.0001 vs. DSS group.

**Supplementary Figure 3. Detection of TGF-β by ELISA in EEN formula.**

TGF-β2 in EEN formula was quantified by ELISA (Cusabio CSB-E14209B). CurveExpert software was used for the curve fit. Data are expressed as mean ± SEM.

**Supplementary Figure 4: Organoid structure analysis.**

**A:** Percentage of immature and mature organoids among counted organoids during culture. **B:** Funnel chart of dead organoids percentage in each condition during culture (top, middle of each funnel bloc). The percentage of dead organoids at day 6 or day 9, considering the maturity category at the previous analysis day (e.g: among 47% of DSS dead organoids at d6 of culture, 49% of them were immature at d3 of culture).

**Supplementary Figure 5: Quantitative PCR of CT, DSS, DM crypts.**

Relative expressions of colonocytes markers (A: Ap1, B: Sis, C: Fab1), Goblet cells (D: Clca), enteroendocrine cells (E: Cgc), and Tuft cells (F: Sucr), Mucins (H: Muc2, I: Muc4, J: Muc3), antimicrobial peptides ( J: Tff3, K: Mmp7) and tight junction protein (L: Ocld), receptor of IgA translocation (M: PigR). Data are expressed as mean ± SEM; At least n>4 mouse per group. Statistics: CT vs DSS, and DM vs DSS. *P-values*: * p<0.05; ** p<0.01; ***p<0.001; ****p<0.0001.

**Supplementary Tables**

**Supplementary Table 1:** **Nutritional composition of Modulen-IBD® and Infatrini Peptisorb® per 100 ml of liquid**.

The percentage of differences is calculated with Modulen IBD® as reference.

**Supplementary Table 2: Gene sequences used for qPCR.**

NCBI design tool was used to design sequences**.**
